# Neonatal Netherton syndrome: Dermoscopic clues for early diagnosis

**DOI:** 10.1016/j.jdcr.2025.11.020

**Published:** 2025-11-21

**Authors:** Suttikit Pikulpol, Parith Wongkittichote, Sanchawan Wittayakornrerk

**Affiliations:** a Division of Dermatology, Department of Medicine, Faculty of Medicine Ramathibodi Hospital, Mahidol University, Bangkok, Thailand; b Division of Genetics, Department of Pediatrics, Faculty of Medicine Ramathibodi Hospital, Mahidol University, Bangkok, Thailand; c Unit of Dermatology, Department of Pediatrics, Faculty of Medicine Ramathibodi Hospital, Mahidol University, Bangkok, Thailand

**Keywords:** bamboo hairs, exfoliative dermatitis, Netherton syndrome, SPINK5, trichorrhexis invaginata

## Introduction

Netherton syndrome (NS) is a rare autosomal recessive disorder caused by loss-of-function mutations in the *SPINK5* gene, which encodes lymphoepithelial Kazal-type-related protease inhibitor (LEKTI).^1^^,^^2^ It is characterized by a triad of congenital erythroderma or exfoliative dermatitis, hair shaft defects—most notably trichorrhexis invaginata (TI)—and atopic diathesis.^1^^,^^3^ The clinical presentation in neonates is often nonspecific, frequently leading to delayed diagnosis or misdiagnosis as other causes of neonatal erythroderma. Detection of TI, or “bamboo hair,” is pathognomonic for NS and provides a key diagnostic clue, although its recognition during the neonatal period remains uncommon.

## Case report

A 30-day-old Thai female neonate was referred to our hospital for evaluation of progressive skin desquamation. She was born at 37 weeks of gestation via vaginal delivery, with a birth weight of 2715 g. Her perinatal course was complicated by moderate hypoxic-ischemic encephalopathy (HIE), for which she received therapeutic hypothermia for 72 hours. On day 6 of life, she developed ventilator-associated pneumonia. Tracheal aspirate cultures grew *Stenotrophomonas maltophilia*, and she was treated with intravenous levofloxacin for 10 days.

Since birth, the infant had diffused fine scaling and peeling of the skin, most prominent on the hands. By day 21 of life, her condition progressed to extensive exfoliative dermatitis. There was no family history of ichthyosis or other dermatologic disorders.

**Fig 1:**
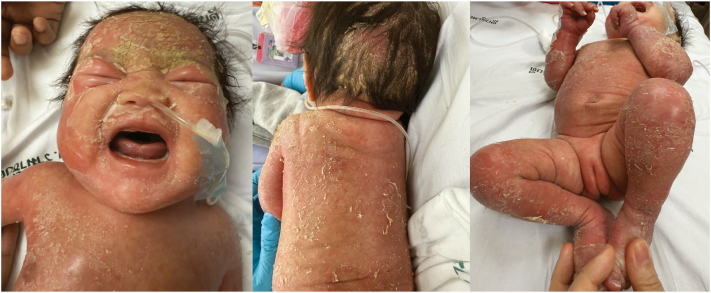
Exfoliative dermatitis with localized alopecia in 30 day-old-female infant.

At a presentation to our hospital on day 30 of life, her vital signs were stable: temperature 36.5 °C, heart rate 160 bpm, respiratory rate 48 breaths/min, and blood pressure 99/60 mmHg. Growth parameters were below the third percentile (weight 2780 g; length 46 cm). Dermatologic examination revealed thick, yellowish, adherent scales over the scalp and face, patchy alopecia, and diffuse erythematous, scaly plaques on the trunk and extremities (Fig 1). Sparse scalp hair was noted, while nails and mucous membranes appeared normal.

**Fig 2:**
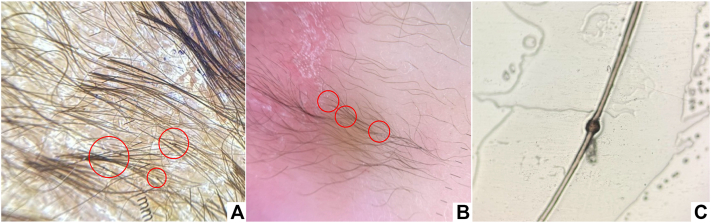
Trichoscopic images show trichorrhexis invaginata (*red circle*) of the scalp hair **(A)** and eyebrows **(B)**. Microscopic image of trichorrhexis invaginata **(C)**.

Given the presence of neonatal exfoliative dermatitis with alopecia, further systemic evaluation was undertaken. A dermoscopic examination of the hair and eyebrows, also known as trichoscopy, revealed hair shaft abnormalities suggestive of TI (Fig 2, *A* and *B*), confirmed by light microscopy as “bamboo hair” (Fig 2, *C*), supporting a clinical diagnosis of Netherton syndrome.

**Fig 3:**
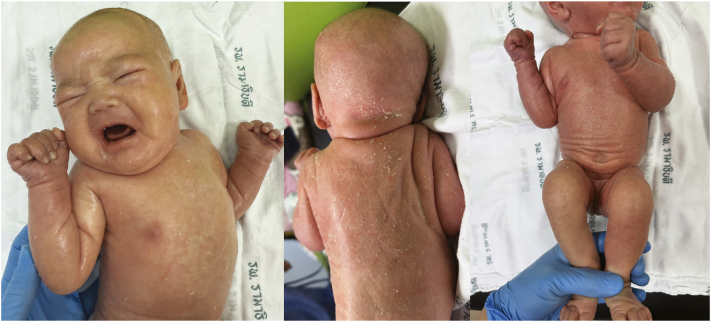
Clinical improvement following treatment.

Laboratory investigations revealed peripheral eosinophilia (absolute eosinophil count of 1600 cells/μL; normal <700/μL), hypernatremia (serum sodium 153 mmol/L; normal 135-145 mmol/L), and elevated serum specific IgE (160 IU/mL; normal <15 IU/mL in infants). Supportive management included intravenous hydration for dehydration, thermal regulation through incubator humidity adjustment, and aggressive topical therapy with emollients (petroleum jelly and liquid paraffin). These interventions led to significant improvement in skin condition, normalization of electrolytes, and stabilization of body temperature (Fig 3). The patient was discharged home after 2 weeks of hospitalization. On follow-up in the outpatient clinic, her skin continued to improve with ongoing emollient therapy, and no significant infectious complications were observed.

**Fig 4:**
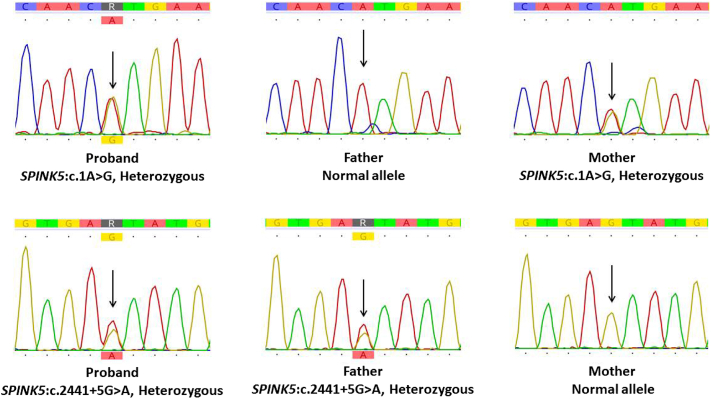
Sanger sequencing of the proband and the parents.

Exome sequencing was performed (the Illumina NovaSeq X Plus platform, South Korea). Variant analysis was guided by Human phenotype ontology terms “erythroderma,” and “ichthyosis,” to prioritize clinically relevant genetic variants. Two heterozygous variants in the *SPINK5* gene were detected: c.1A>G (p.Met1?) and c.2441+5G>A. The variants are present in a *trans* configuration, with each parent identified as a heterozygous carrier through Sanger sequencing as shown in Fig 4.

## Discussion

TI is considered pathognomonic for NS.^2^, ^3^, ^4^ The onset of hair shaft abnormalities in NS is variable and typically becomes more apparent after the first year of life, when hair growth is more developed.^4^, ^5^, ^6^, ^7^, ^8^ However, a few case reports have documented earlier detection during the neonatal or early infantile period. For instance, Okulu et al^9^ identified TI in a neonate with genetically confirmed NS using light microscopy, while Di Nora et al^10^ reported videodermoscopy findings of TI in a 1-month-old infant. These observations suggest that although TI often develops progressively, subtle hair shaft deformities may already be present during the neonatal period. Conversely, Burk et al^6^ observed a delayed onset of hair abnormalities—hair shafts appeared normal at ages 2 and 4 years but later demonstrated TI, which was identified by dermoscopy at 7 years of age. Nevertheless, the absence of TI in early life does not preclude a diagnosis of NS.

In our patient, generalized exfoliative dermatitis, growth retardation, and elevated serum IgE were observed—findings commonly associated with NS. However, these clinical features overlap with other causes of neonatal erythroderma, including congenital ichthyoses, severe atopic dermatitis, Omenn syndrome, and infections such as staphylococcal scalded skin syndrome or candidiasis.^5^ A distinguishing feature in this case was the early detection of hair shaft anomalies through dermoscopy, which played a pivotal role in narrowing the differential diagnosis.^5^, ^6^, ^7^, ^8^

Dermoscopy, a noninvasive and bedside-accessible tool, revealed abnormalities in both scalp and eyebrow hair, prompting further examination with light microscopy. However, its role in neonates remains underrecognized. Previous reports have rarely highlighted dermoscopy as a frontline diagnostic tool in this age group, despite its advantages: it is rapid, noninvasive, widely available, and feasible at the bedside. In our patient, dermoscopy provided a critical diagnostic clue that would otherwise have been missed, as skin biopsy in neonates is less desirable and hair sampling may be limited.

In our patient, exome sequencing identified compound heterozygous variants in *SPINK5*. The initiation codon change (c.1A>G) is predicted to cause loss of function due to decreased protein synthesis, as reported by et al.^11^ In addition, the splice-site variant (c.2441+5G>A) was predicted to cause splicing aberration using SpliceAI, as described by Jaganathan et al.^12^ Both variants are likely to result in loss of function of the *SPINK5* protein, thereby confirming the diagnosis of Netherton syndrome.

This case underscores the potential of dermoscopy to bridge a diagnostic gap in neonatal erythroderma. To our knowledge, only a limited number of cases in the literature have emphasized the utility of dermoscopy in the neonatal period for diagnosing hair shaft abnormalities. Early recognition of NS is clinically important, as affected neonates are at high risk of dehydration, electrolyte imbalance, recurrent infections, and failure to thrive. Early supportive measures, as demonstrated here, may improve outcomes and prevent complications.

## Conflicts of interest

None disclosed.
